# Time series analysis of comprehensive maternal deaths in Brazil during the COVID-19 pandemic

**DOI:** 10.1038/s41598-024-74704-x

**Published:** 2024-10-14

**Authors:** Mary Catherine Cambou, Hollie David, Corrina Moucheraud, Karin Nielsen-Saines, Warren Scott Comulada, James Macinko

**Affiliations:** 1 University of California, Los Angeles, USA; 2 New York University, New York, USA

**Keywords:** Maternal mortality, Comprehensive maternal deaths, Time series analysis, Brazil, COVID-19, Infectious diseases, Public health

## Abstract

The effects of the COVID-19 pandemic on comprehensive maternal deaths in Brazil have not been fully explored. Using publicly available data from the Brazilian Mortality Information (SIM) and Information System on Live Births (SINASC) databases, we used two complementary forecasting models to predict estimates of maternal mortality ratios using maternal deaths (MMR) and comprehensive maternal deaths (MMRc) in the years 2020 and 2021 based on data from 2008 to 2019. We calculated national and regional standardized mortality ratio estimates for maternal deaths (SMR) and comprehensive maternal deaths (SMRc) for 2020 and 2021. The observed MMRc in 2021 was more than double the predicted MMRc based on the Holt-Winters and autoregressive integrated moving average models (127.12 versus 60.89 and 59.12 per 100,000 live births, respectively). We found persisting sub-national variation in comprehensive maternal mortality: SMRc ranged from 1.74 (95% confidence interval [CI] 1.64, 1.86) in the Northeast to 2.70 (95% CI 2.45, 2.96) in the South in 2021. The observed national estimates for comprehensive maternal deaths in 2021 were the highest in Brazil in the past three decades. Increased resources for prenatal care, maternal health, and postpartum care may be needed to reverse the national trend in comprehensive maternal deaths.

**Supplementary Information:**

The online version contains supplementary material available at 10.1038/s41598-024-74704-x.

## Introduction

Maternal mortality, defined as death during pregnancy, childbirth or within 42 days postpartum or termination (regardless of cause^1^), remains a leading cause of death among women globally^2^. The majority of maternal deaths are due to postpartum hemorrhage, infection, and hypertensive disorders of pregnancy (HDP), the latter of which is the cornerstone of modern prenatal care^3^. There is growing interest in comprehensive maternal deaths as a marker of maternal care quality, defined as the combined grouping of maternal deaths and late maternal deaths, up to one year postpartum^1^. While the 50% reduction in the global maternal mortality ratio (MMR) between 1990 and 2015 is a testament to the collective effort worldwide to improve maternal health, this progress fell short of the Millennium Development Goal (MDG) of a 75% reduction in the global MMR over this period^4^. The new Sustainable Development Goal (SDG) target calls for a global MMR less than 70 maternal deaths per 100,000 live births by 2030^5^; it is currently 223 maternal deaths per 100,000 live births^6^.

The COVID-19 pandemic slowed the progress made in MMR reduction in many countries, including in Brazil, although the true extent of this setback is unknown^6^. Pandemics are destabilizing events with both direct and indirect immediate and long-term effects. Not only are pregnant persons at higher risk of severe COVID-19^7^, but the pandemic itself impacted their ability to access health services in a timely fashion. Preventive services in Brazil, including prenatal care, were highly disrupted by the pandemic^8^, including the identification and treatment of HDP, a leading cause of maternal mortality and morbidity worldwide^9^. SARS-CoV-2, the virus responsible for COVID-19, is a trigger of HDP, further complicating maternal health outcomes during the pandemic^10,11^. In addition, hospital resources normally designated for prenatal care were diverted to accommodate the unprecedented strain that was placed on the healthcare system as a result of the COVID-19 surges, particularly in 2021^12^.

Before to the pandemic, Brazil made great strides in improving maternal care quality through several designated public health programs^13–15^. While maternal mortality in Brazil is relatively higher compared to similar middle-income countries, the MMR decreased from an estimated 84.5 per 100,000 live births in 1990 to 65.4 per 100,000 live births in 2015^16^. However, studies at the regional and state levels in Brazil demonstrated an unexpected rise in MMR during the first year of the COVID-19 pandemic^17^, likely due to a combination of COVID-19-related maternal deaths, and increasing barriers to perinatal care access impacting maternal care quality^18^.

While several studies at different time points have investigated the impact of the pandemic on maternal deaths in Brazil^17,19–23^ the excess burden of COVID-19 on comprehensive maternal deaths in Brazil during the first two years of the pandemic has not been fully characterized. In order to estimate excess maternal mortality in Brazil in 2020 and 2021 due to COVID-19, we used a combination of (1) two forecasting methods, the non-seasonal Holt-Winters exponential smoothing (HES) model and autoregressive integrated moving average (ARIMA) model, to predict the MMR for maternal deaths, and for comprehensive maternal deaths (MMRc) in 2020 and 2021 based on time series data from 2008 to 2019, and (2) the Standardized Mortality Ratio for maternal deaths (SMR) and comprehensive maternal deaths (SMRc) at the national and regional levels for 2020 and 2021. We hypothesized that (1) there was a significant relative increase in MMR and MMRc in 2020 and 2021 compared to the projected estimates, and (2) maternal deaths attributed to COVID-19 as the direct cause accounted for the majority of official maternal and comprehensive maternal deaths according to the Ministry of Health. This study serves to broaden the literature through the investigation of forecasting models with comparison to SMR estimates of both maternal and comprehensive maternal deaths using the most current publicly available data.

## Methods

### Data source

Brazil consists of five macro-regions: the North, Northeast, Central-West, Southeast, and South. The 26 states and the Federal District, for a total of 27 federative units, form the macro-regions. The states are further divided into 5,570 municipalities, each with its own autonomous local government, including a mayor, municipal chamber, and health secretariat responsible for local management and delivery of healthcare in facilities other than hospitals (which are mostly state or federally owned)^24^. While healthcare is a constitutional right in Brazil and the public health system (Sistema Único de Saúde, SUS) is available free of charge in most settings, income, social and health inequities are widespread throughout Brazil, with persistent regional differences in employment, educational attainment, and access to basic services^25,26^.

The Brazilian Mortality Information System (SIM)^27^ is a national database for microdata on deaths reported at the municipal level^28^. Data are transferred from municipalities to states, and subsequently managed at the national level by the Brazilian Ministry of Health. We used aggregate data to estimate (1) maternal deaths, defined as the number of annual deaths from any cause during pregnancy and labor, up to 42 days of termination of pregnancy, and (2) late maternal deaths, defined as the number of annual deaths from any cause related to pregnancy or management more than 42 days following termination of pregnancy and up to one year postpartum^1^. The Brazilian Information System on Live Births (SINASC)^29,30^ is a national database for all live births reported at the municipal level^29^. Aggregate data at the state and national levels were collected via the Integrated Health Surveillance (IVIS) platform^31^.

Beginning in January, 2020, the publicly-available Sistema de Informação da Vigilancia Epidemiológica da Gripe (SIVEP-Gripe) database began tracking all COVID-19 hospitalizations in Brazil through the Unified Health System platform (DATASUS)^32^. The SIVEP-Gripe surveillance system was established in 2009 following the H1N1 influenza pandemic^33^. Since its inception, the database has served as the national surveillance system for influenza and other respiratory viruses of clinical concern^34^. Following the initial identification of SARS-CoV-2, the Brazilian Ministry of Health required notification of both suspected and confirmed cases by polymerase chain reaction (PCR) testing (the gold standard), and now antigen (Ag) testing^35^. Both public and private hospitals are required by law to report on COVID-19 hospitalizations via the electronic database within 24 hours of a suspected case^35^. Data collected include geographic location, medical co-morbidities, pregnancy and postpartum status, hospital course complications, and outcomes, including death. The annual databases, including reporting details and the data dictionary, are made available through the DATASUS platform^32^. The data are reviewed and cleaned weekly by the Ministry of Health National Immunization Program.

### Statistical analysis

We used national aggregate data from the SIM^27^ and SINASC^30^ databases to calculate the annual observed MMR and MMRc per 100,000 live births in Brazil from 2008 to 2021.

$$Annual\;MMRc = \frac{{Observed\;comprehensive\;maternal\;deaths\;in\;a\;given\;year \left( {from\;SIM} \right)}}{{Total\;live\;births\;in\;a\;given\;year \left( {from\;SINASC} \right)}} \times 100,000$$

We used the non-seasonal HES model, a forecasting time series method, based on time series data of MMR and MMRc from 2008 to 2019, to predict MMR and MMRc, respectively, for 2020 and 2021. The HES model is often used for forecasting time series^18,36,37^. It builds on the simple exponential smoothing (SES) method:

$${L}_{t}=\alpha {y}_{t}+\left(1-\alpha \right){L}_{t-1}$$

where “y_t_ is the value at current time step t, L_t_ is the level estimate for t, L_t-1_ is the previous level estimate, and $\alpha$ is a smoothing constant”^37^.

The HES model is considered a second exponential smoothing method, as the approach incorporates trend into the SES model:

$${F}_{t+k}={L}_{t}+k{T}_{t}$$

where “Lt is the level estimate for time t, k is the number of forecasts into the future, and T_t_ is the trend at time t”^37^. We used the iterative process to define the smoothing parameters^38,39^. To account for potential data quality variance, 12 data points were used. Predicted MMR and observed MMR with 95% confident intervals (CIs) from 2008 to 2021 were plotted, and repeated with MMRc estimates. We then conducted a sensitivity analysis in an effort to control for over-fitting with an ARIMA model and test whether the findings were sensitive to the HES model^40,41^. The ARIMA model parameters (p: lag order, d: degree of differencing, q: order of moving average) were selected based on minimizing AIC and BIC^42^.

Next, we plotted the maternal deaths at the national level, from 2008 to 2021. To explore geographic variation, maternal deaths were categorized and plotted by the five macro-regions in Brazil: the poorer North and Northeast, and wealthier South, Southeast, and Central-West. We calculated national and regional standardized mortality ratio (SMR) estimates^43^ in 2020 and 2021, using maternal deaths in 2019 as the reference^44^.

$$SMR=\frac{Observed\;deaths\; (in\; a\; study\; population)}{Expected\; deaths\; (in\; a\; study\;population)}$$

We then calculated the national regional SMR estimates for comprehensive maternal deaths (SMRc), defined as the sum of maternal deaths and late maternal deaths, due to direct or indirect obstetric causes, up to one year postpartum or one year post-termination^1^. The 95% CIs for each SMR and SMRc estimates were calculated using the Vandenbroucke method^45^. Statistical analysis was performed with STATA version 16. This study used de-identified, publicly available data through the Unified Health System platform (DATASUS) through the Brazilian Ministry of Health. The methods were carried out in accordance with the UCLA IRB. The protocols were deemed IRB exempt per review by the UCLA Office of Human Research Protection Program (IRB #23-000145).

## Results

From January 1, 2020, to December 31, 2021, there were an estimated 4,995 maternal deaths, and 5,541 comprehensive maternal deaths in Brazil, with the majority occurring in 2021. There was initial increase in maternal deaths during the H1N1 influenza pandemic^46^, although the MMR and MMRc trended down after 2009. Among maternal deaths in this period, 1,336 were attributed to COVID-19 in pregnancy, with the majority (79.2%) occurring in 2021.

**Fig. 1:**
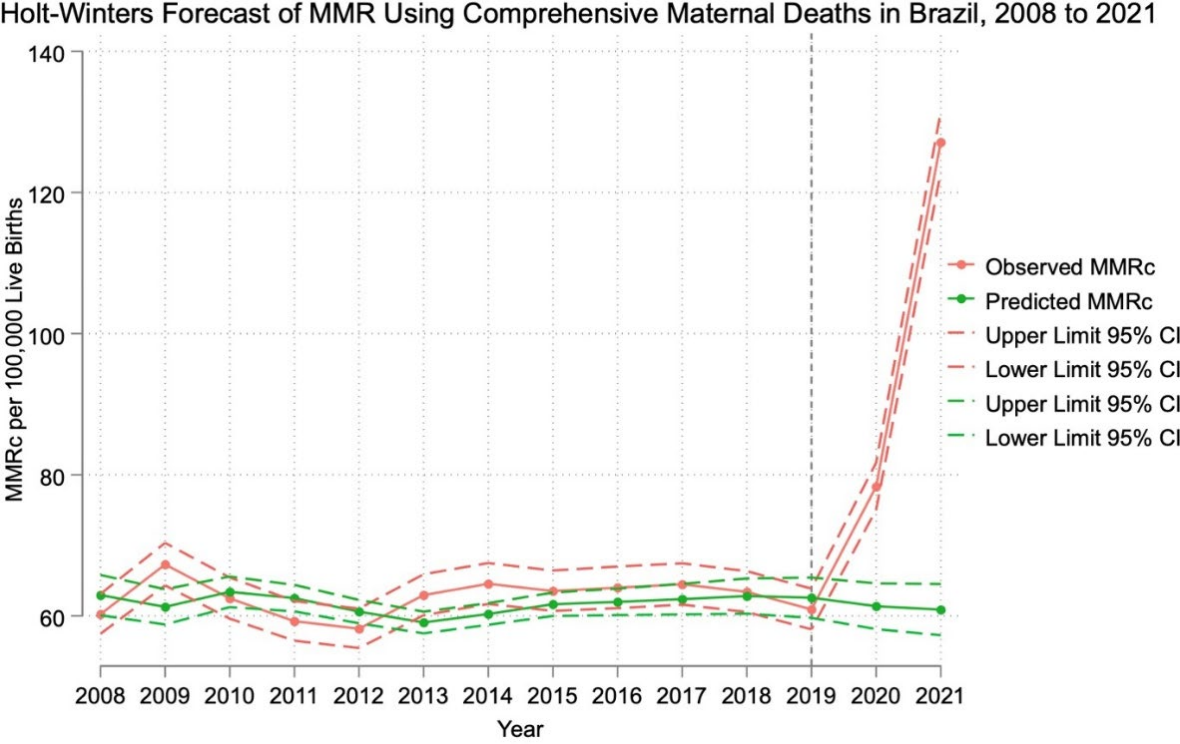
Holt-Winters forecast of predicted MMRc compared to observed MMRc in Brazil from 2008 to 2021. National aggregate data from the SIM and SINASC databases were used to calculate the annual observed MMRc per 100,000 live births in Brazil from 2008 to 2021. A non-seasonal Holt-Winters exponential model based on time series data of MMRc from 2008 to 2019 was used to predict MMRc for 2020 and 2021. Smoothing parameters were selected by an iterative process to minimize the in-sample sum-of-squared prediction error.

Figure 1 shows the Holt-Winters forecast of predicted MMRc compared to observed MMRc in Brazil from 2008 to 2021. While the model predicted a downward slope from 2019 to 2021, the observed MMRc estimates in 2020 and 2021 increased from 78.31 to 127.12 per 100,000 live births, respectively. The observed MMRc was more than double the predicted MMRc in 2021 based on the Holt-Winters forecast estimate (127.12 versus 60.89 per 100,000 live births). Supplemental Fig. 1 shows the Holt-Winters forecast of predicted MMR using maternal deaths only, showing a similar trend.

**Fig. 2:**
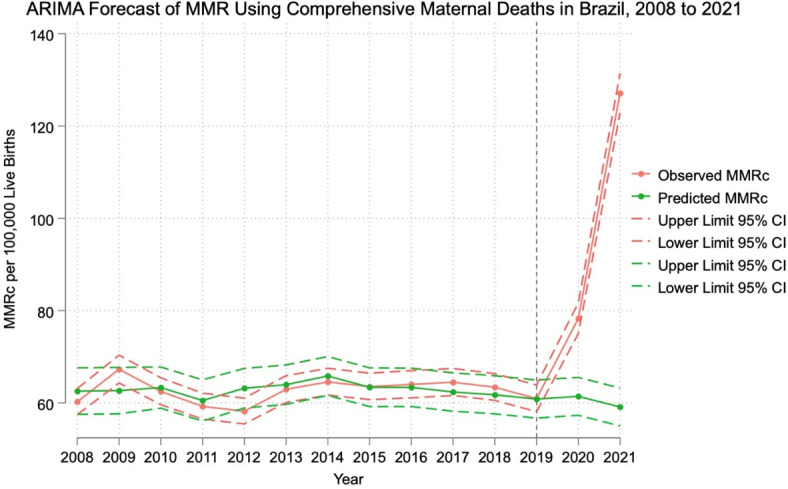
ARIMA forecast of predicted MMRc compared to observed MMRc in Brazil from 2008 to 2021. National aggregate data from the SIM and SINASC databases were used to calculate the annual observed MMRc per 100,000 live births in Brazil from 2008 to 2021. ARIMA model parameters (0,0,2) were selected based on minimizing AIC and BIC.

Figure 2 shows the ARIMA forecast of predicted MMRc compared to observed MMRc in Brazil from 2008 to 2021. The ARIMA model predicted MMRc estimates of 61.43 and 59.12 in 2020 and 2021, respectively. The MMR estimates were comparable to those predicted by the Holt-Winters forecast. The observed MMR was again more than double the predicted MMR in 2021 based on the ARIMA estimate (113.18 vs 58.83 per 100,000 live births). Supplemental Fig. 2 shows the ARIMA forecast of predicted MMR using maternal deaths only, showing a similar trend.

**Fig. 3:**
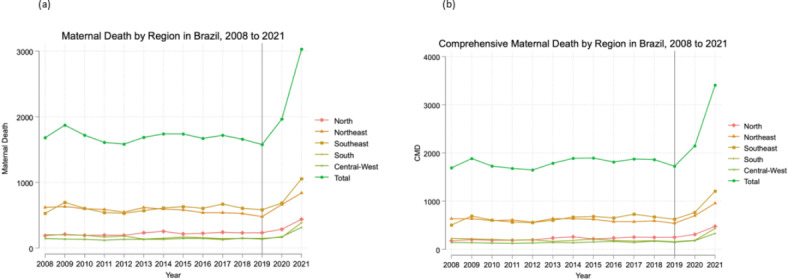
(**a**) Maternal deaths^a^ at the national and regional levels^b^ from 2008 to 2021. (**b**) Comprehensive maternal deaths^c^ at the national and regional levels^d^ from 2008 to 2021. a. Defined according to the WHO as death during pregnancy, childbirth or within 42 days postpartum or termination, regardless of cause. b. To explore geographic variation from 2008 to 2021, maternal deaths were categorized and plotted by the five macro-regions in Brazil: North, Northeast, South, Southeast, and Central-West. c. Defined according to the WHO as the sum of maternal deaths and late maternal deaths. d. To explore geographic variation from 2008 to 2021, comprehensive maternal deaths were categorized and plotted by the five macro-regions in Brazil: North, Northeast, South, Southeast, and Central-West.

Figure 3 shows the maternal deaths (a) and comprehensive maternal deaths (b) from 2008 to 2021 at the national and regional levels. Geographic disparities presents prior to the pandemic persisted across the five macro-regions: the highest number of maternal deaths and comprehensive maternal deaths were concentrated in the Northeast (consisting of the states of Alagoas, Bahia, Ceará, Maranhão, Paraíba, Pernambuco, Piauí, Rio Grande do Norte, and Sergipe) and the Southeast (consisting of the states of Espiríto Santo, Minas Gerais, Rio de Janeiro and São Paulo), while the Central-West (consisting of the states of Goiás, Mato Grosso, Mato Grosso do Sul and Distrito Federal) had the lowest. This trend continued during the first two years of the pandemic, with the highest recorded maternal deaths (1,055) and comprehensive maternal deaths (1,205) observed in the Southeast in 2021.

The observed maternal deaths increased from 2020 to 2021 across all macro-regions. Table 1 compares maternal deaths, estimated percentage due to COVID-19, and SMR at the national and regional levels from 2019 to 2021. In 2020, there were 1,965 maternal deaths, of which approximately 14.10% were due to COVID-19. In 2021, the maternal deaths increased to 3,030, of which an estimated 34.95% were attributed to COVID-19. The proportion of maternal deaths due to COVID-19 in 2021 ranged from 24.34% in the Northeast to 42.04% in the Central-West region. The national SMR estimates for maternal deaths were 1.25 (95% CI 1.19–1.30) in 2020 and 1.92 (95% CI 1.85–1.99) in 2021. The SMR estimates ranged from 1.75 (95% CI 1.63–1.87) in the Northeast, to 2.62 (95% CI 2.36–2.88) in the South during the second year of the pandemic. Table 2 compares comprehensive maternal deaths, and SMRc at the national and regional levels from 2019 to 2021. The national SMRc estimates for comprehensive maternal deaths were 1.23 (95% CI 1.17–1.29) in 2020 and 1.96 (95% CI 1.89–2.03) in 2021. There were 3,403 total comprehensive maternal deaths in 2021, with the highest SMRc in the South Region (2.70, 95% CI 2.45–2.96).

**Table 1:** Comparison of maternal deaths^a^ and SMR^b^ at the national and regional levels, 2019 to 2021.

Year	Region	Maternal deaths	COVID-19 death in hospital	% Due to COVID-19	SMR (95% CI)^c^
2019	Total	1,576	N/A	N/A	Reference
2019	North	233	N/A	N/A	Reference
2019	Northeast	478	N/A	N/A	Reference
2019	Southeast	582	N/A	N/A	Reference
2019	South	147	N/A	N/A	Reference
2019	Central-West	136	N/A	N/A	Reference
2020	Total	1,965	277	14.10	1.25 (1.19, 1.30)
2020	North	285	36	12.63	1.22 (1.08, 1.37)
2020	Northeast	662	82	12.39	1.38 (1.28, 1.49)
2020	Southeast	685	113	16.50	1.17 (1.09, 1.27)
2020	South	162	16	9.88	1.10 (0.93, 1.27)
2020	Central-West	171	39	22.81	1.26 (1.07, 1.45)
2021	Total	3,030	1,059	34.95	1.92 (1.85, 1.99)
2021	North	438	132	30.14	1.88 (1.70, 2.06)
2021	Northeast	838	204	24.34	1.75 (1.63, 1.87)
2021	Southeast	1,055	432	40.95	1.81 (1.70, 1.92)
2021	South	385	159	41.30	2.62 (2.36, 2.88)
2021	Central-West	314	132	42.04	2.31 (2.05, 2.56)

^a^Defined according to the WHO as death during pregnancy, childbirth or within 42 days postpartum or termination, regardless of cause.

^b^SMR = (Observed deaths/Expected deaths), using 2019 as the reference.

^c^95% CI calculated using the Vandenbroucke method.

**Table 2:** Comparison of comprehensive maternal deaths^a^ and SMRc^b^ at the national and regional levels, 2019 to 2021.

Year	Region	Comprehensive maternal deaths	SMRc (95% CI)^c^
2019	Total	1,736	Reference
2019	North	250	Reference
2019	Northeast	546	Reference
2019	Southeast	627	Reference
2019	South	161	Reference
2019	Central-West	152	Reference
2020	Total	2,138	1.23 (1.17, 1.29)
2020	North	310	1.24 (1.10, 1.38)
2020	Northeast	697	1.28 (1.18, 1.37)
2020	Southeast	763	1.22 (1.13, 1.30)
2020	South	187	1.16 (1.00, 1.33)
2020	Central-West	181	1.19 (1.02, 1.36)
2021	Total	3,403	1.96 (1.89, 2.03)
2021	North	479	1.92 (1.74, 2.09)
2021	Northeast	954	1.74 (1.64, 1.86)
2021	Southeast	1,205	1.92 (1.81, 2.03)
2021	South	435	2.70 (2.45, 2.96)
2021	Central-West	330	2.17 (1.94, 2.41)

^a^Defined according to the WHO as the sum of maternal deaths and late maternal deaths.

^b^SMR = (Observed deaths/Expected deaths), using 2019 as the reference.

^c^95% CI calculated using the Vandenbroucke method.

## Discussion

Consistent with our hypothesis, there was a significant relative increase in MMR and MMRc in Brazil in 2020 and 2021 compared to the projected estimates based on both the HES and ARIMA models. Guimarães and colleagues estimated a 40% increase in excess maternal mortality in 2020 based on a Poisson model with robust variance, slightly higher than our estimate^19^. Scheler and colleagues also demonstrated a relative increase in maternal deaths in Brazil during the first half of 2021 compared to 2020, reporting a two-fold increase in the mortality rate among hospitalized pregnant and postpartum individuals with COVID-19^23^. However, we used the most current SIM and SINASC data, representing the most up-to-date mortality and live birth data available in Brazil. Using three forecasting methods based on quarterly data from 1996 to 2021 to compare observed and predicted MMR, Cañedo and colleagues found that Brazil had the highest MMR in the 2^nd^ quarter of 2021, with 197 per 100,000 live births, compared to 61 and 60 per 100,000 live births using a Holt-Winters and ARIMA model, respectively^21^. Similarly, we found that the observed MMR in Brazil was more than double the predicted MMR in 2021 based on our Holt-Winters forecast and ARIMA model estimates. Notably, we also showed that the observed MMRc was more than double the predicted MMRc estimates in 2021 for both models using comprehensive maternal deaths in Brazil, on which the literature is scarce.

The sharp rise in maternal deaths and comprehensive maternal deaths in Brazil during the pandemic mirrors the trend in several countries, including the United States: in 2021, there were an estimated 1,205 maternal deaths, compared to 754 deaths in 2019^47^. However, the estimated MMR in the United States in 2021 was 32.9 per 100,000 live births, representing less than one-third the MMR in Brazil in 2021. While there was a slight increase in MMR in the United States and Brazil during the H1N1 influenza pandemic^46,48^, this stark difference in the 2021 estimates between the two countries highlight the severity of maternal mortality in Brazil during the COVID-19 pandemic, where the 2021 MMR is the highest observed in the country in the past three decades^49^. Furthermore, we used a conservative definition for the MMR estimates, excluding garbage codes. Our observed MMR estimate in 2015, for example, was lower than that from the Global Burden of Disease (GBD) Study (57.59 vs 65.4 per 100,000 live births), although the GBD redistributed cause-specific and garbage ICD-10 codes to capture maternal deaths that were not counted as official^16^.

We found persisting sub-national variation in maternal deaths and comprehensive maternal deaths, with some regions experiencing far higher maternal deaths and comprehensive maternal deaths than others both before and during the pandemic. At the state-level, Carvalho-Sauer and colleagues demonstrated that the 2020 MMR estimate in Bahia state based on a Holt-Winters forecast of time series data from 2011 to 2019 was 49 per 100,000 live births (95% CI 38 to 59 per 100,000 live births), significantly lower than the observed MMR of 78 per 100,000 live births that year^18^. Orellana and colleagues demonstrated that in the North, South and Central-West regions, excess maternal mortality was not significant during 2020^17^. However, our estimates used the most current data, and suggest that all macro-regions except for the South had significantly higher maternal deaths in 2020 compared to 2019. Furthermore, we included comprehensive maternal deaths as well, which showed a similar and more striking trend.

Another paper by Orellana and colleagues used a generalized additive model with a quasi-poisson approach to estimate excess maternal mortality in Brazil and across the regions^20^. Using this approach, the team estimated 39% and 100% excess maternal deaths in 2020 and 2021, respectively, compared to 25% and 92% according to SMR estimates generated in our analysis. Not only did Orellana and colleagues use a different methodological approach, but we used a more conservative definition of maternal death without additional ICD-10 codes, which may explain the differences across our studies.

Surprisingly, direct COVID-19-related maternal deaths did not account for the majority of maternal deaths as we hypothesized, although the proportion due to COVID-19 more than doubled from 2020 to 2021. Guimarães and colleagues demonstrated that COVID-19-related maternal deaths in 2020 did not account for all excess maternal mortality at the national level, pointing to indirect causes of the pandemic on maternal care quality, including interruption of prenatal care^19^. In the United States, a Government Accountability Office report from October, 2022 on maternal deaths during the pandemic to congressional addresses found that COVID-19 was the cause in 25% of maternal deaths in 2020 and 2021, but the pandemic worsened disparities affecting access to care, transportation, and living environment, leading to downstream effects on maternal health^50^. The increase in comprehensive maternal deaths we documented across the macro-regions in 2021 not only highlights the disruptions in access to health care services resulting from the pandemic, but also points to delayed complications from COVID-19, including heart failure and myocardial infarction^51^. Further research is needed to characterize the etiology of the excess comprehensive maternal deaths, and increased resources are likely needed during the postpartum period.

While the highest proportions of maternal deaths and comprehensive maternal deaths were concentrated in the Northeast and Southeast, the macro-region with the largest population, the South and Central-West regions witnessed the largest relative increase in SMR and SMRc in 2021.The high MMR in the Southeast, North, and Northeast are consistent with historical maternal mortality data, driven by economic inequities and structural racism^49,52^. The increase in SMR in the South and Central-West was unexpected^20^, and points to the overwhelming healthcare strain throughout the country resulting from the Gamma surge during the second year of the pandemic^20,53^. Orellana and colleagues found that while there was variation by region and maternal age group during the first two years of the pandemic, there was a significant increase in maternal deaths in the 35 to 49 age group across all five regions during the March to June period of 2021^20^. The South has a relatively higher proportion of pregnancies of advanced maternal age compared to the other regions^54^, which is a known risk factor for increased maternal morbidity among pregnant persons with COVID-19^55^. Furthermore, the increase in SMR and SMRc in the South in 2021 coincided with the SARS-CoV-2 variant Gamma becoming the primary circulating variant of concern^20^.

The Gamma surge during the first half of 2021 in Brazil lead to an unprecedented strain on the healthcare system in Brazil^56^, with profound implications for maternal care quality and the treatment of COVID-19 in pregnancy. Giovanetti and colleagues demonstrated that the Gamma variant accounted for over 95% of cases in the country during the first half of 2021^57^. During the first three months of 2021, 15 states reported >90% ICU bed capacity, and several states reported 100% ICU bed capacity^56,58^, leading to denial of or delays in ICU-level care for thousands of Brazilians^56^. A qualitative study by Diniz and colleagues provide context for how such disruptions may have affected maternal health by documenting the frustration of family members attempting to access care for their pregnant or postpartum relatives who ultimately died from COVID-19^59^. Many of them cited multiple unsuccessful attempts to access outpatient care before hospitalization, the dismissal of COVID-related symptoms, and significant delays in both hospitalizations and ICU admission due to health care system strain when COVID-19 progressed to severe disease^59^. While maternal deaths directly attributed to COVID-19 increased from the first to the second year of the pandemic, the significant increase in excess maternal mortality resulting from non-COVID causes in 2021 highlights the negative impact of the pandemic on maternal care quality.

## Limitations

Our study has several limitations. First, MMR estimates are notoriously difficult to capture due to inaccurate reporting of maternal death^60^. While SIM uses the standardized definition of maternal death up to 42 days after delivery, maternal death is likely under-reported worldwide with increasing duration of postpartum days. Several studies have documented undercounting and regional differences in mortality data via SIM compared to the National Statistics Office (IBGE)^61,62^. While mortality estimates have improved over the past decade^63^, there are concerns about completeness, particularly from rural states and the Northern region^61^. However, the Brazilian Ministry of Health has invested significant resources into improving the collection and dissemination of maternal mortality data. Since 2008, an investigation into the causes of death for any woman of reproductive age is led by a city-specific Death Surveillance Reference Team, which is then entered into the SIM system within four months, and registered by the Municipal Department of Health^49^. This formal process has greatly improved the quality of data for maternal deaths and late maternal deaths. Regardless, we suspect that the national and regional MMR, SMR and SMRc estimates in 2020 and 2021 are lower than the true values.

Second, estimates of maternal deaths due to COVID-19 range across studies^17,19,23^. We used the most conservative definition of COVID-19 in pregnancy, based on laboratory confirmation with a positive PCR or antigen test. Therefore, while our study shows a significant increase in MMR and SMR due to COVID-19 in pregnancy, these values likely underestimate the true burden of COVID-19 on maternal mortality in Brazil due to racial and economic disparities in laboratory testing^64^.

Third, forecasting depends on historical time series points, therefore the accuracy of the predictions rely on both the quantity and quality of the time series data. We addressed this by using 12 data points to forecast two MMR and MMRc values; and used the HES method, which is robust and frequently used for forecasting in surveillance^65^. While time series models are vulnerable to over-fitting^66^, we addressed this by running a sensitivity analysis with an ARIMA model, which produced similar MMR and MMRc estimates.

Fourth, the significance of associations in a time series observational study should be interpreted with caution since the possibility of confounding cannot be excluded. However, given the increased risk of mortality with SARS-CoV-2 infection in pregnancy and its impact on maternal health care delivery, it is reasonable to assume that the increased MMR in Brazil in 2020 and 2021 was driven primarily by COVID-19.

Last, there was a slight reduction in annual fertility rates following the Zika virus (ZIKV) epidemic in Brazil in 2015. This may have resulted in less pressure on prenatal and maternal health care programs, thereby resulting in an improvement in MMR and MMRc estimates. However, this would be difficult to measure, and the MMR and MMRc estimates remained relatively stable from 2015 to 2017.

## Conclusions

Our findings add to the global maternal health literature, and will be of interest to those working in health services, particularly in the context of middle-income countries with universal healthcare coverage. As maternal mortality is often used as a proxy for health services availability, this paper provides insight into both the direct and indirect effects of COVID-19 on healthcare infrastructure for pregnant persons in Brazil. There is a paucity of studies on comprehensive maternal deaths in Brazil, an important indicator of the delayed complications of a SARS-CoV-2 infection in pregnancy on maternal outcomes, which our paper addresses.

During the first two years of the COVID-19 pandemic, there were over 5,500 comprehensive maternal deaths in Brazil. Using two complementary forecasting models, we estimated that the observed MMRc was more than double the predicted MMR in 2021. Excess maternal deaths and comprehensive maternal deaths at the national level increased by over 92% during the second year of the pandemic, with over one-third of the maternal deaths in 2021 due directly to COVID-19 in pregnancy or in the postpartum period. While there was significant geographic variation in SMR and SMRc estimates, excess maternal mortality surpassed 74% in all macro-regions in 2021, coinciding with the Gamma surge during the first half of 2021^53^.

The observed MMR and MMRc estimates in Brazil in 2021 are the highest in the past three decades^52^. A paper by Carvalho-Sauer and colleagues using an interrupted time series analysis of maternal mortality rates in Brazil suggests an improvement in 2022, likely due to less severe COVID-19 in pregnancy resulting from a combination of hybrid immunity and vaccination^67,68^ (and a possible lessening of the severe impacts to the health system). The long-term effects of previous SARS-CoV-2 infection and subsequent development of HDP in pregnancy remain unclear^10,11,69,70^. Furthermore, it is unknown how quickly the health care system in Brazil will recover in order to meet the SDG of MMR <70 per 100,000 live births by the year 2030^4^. Increased resources, including continued investment in Brazil’s Family Health Strategy program of community health workers^15^, may be needed to strengthen the delivery of high-quality perinatal and notably postpartum care in vulnerable regions. Municipalities and states in the North and Northeast with high MMR and MMRc pre-COVID-19 should be of particular focus.

Another strategy to protect against maternal deaths and comprehensive maternal deaths includes ongoing government support for national and regional vaccination campaigns, with an emphasis on pregnant and postpartum individuals, as well as other persons capable of pregnancy. A study by Santos and colleagues to evaluate the impact of the national vaccination campaign in Brazil in 2021 estimates that 875,846 cases of severe COVID-19 and 303,129 deaths among adults were averted as a result of the roll-out^71^. Furthermore, the paper by Carvalho-Sauer and colleagues showed an improvement in MMR following the COVID-19 vaccination campaign directed toward pregnant persons^67^. While 80% of Brazil had received at least one dose of the COVID-19 vaccine by the beginning of 2022^72^, vaccination rates among pregnant persons remain subpar, driven largely by vaccine hesitancy, including that of some obstetric providers^73^. As of January, 2023, the Brazilian Federation of Gynecology and Obstetrics Associations recommend the CoronaVac and BNT162b2 mRNA vaccines for all pregnant and lactating persons^74^, representing a major step in the implementation and uptake of the COVID-19 vaccine in pregnancy. Targeted public health campaigns to encourage COVID-19 vaccine uptake among pregnant, postpartum and lactating persons, as well as women and persons of reproductive age, has the potential to reduce MMR and MMRc in Brazil.

## Electronic supplementary material

Below is the link to the electronic supplementary material.

Supplementary Material 1

## Data Availability

Datasets analyzed during this study are publicly available through the Brazilian Ministry of Health. Data on maternal deaths are currently available via the Brazilian Mortality Information System (SIM): http://tabnet.datasus.gov.br/cgi/tabcgi.exe?sim/cnv/obt10uf.def. Live birth data are currently available via the Brazilian Information System on Live Births (SINASC): http://tabnet.datasus.gov.br/cgi/deftohtm.exe?sinasc/cnv/nvuf.def. Aggregate national and state data are also available via the Integrated Health Surveillance (IVIS) platform: http://plataforma.saude.gov.br/natalidade/nascidos-vivos/. COVID-19 data are available through the publicly-available Sistema de Informação da Vigilancia Epidemiológica da Gripe (SIVEP-Gripe) database via the Unified Health System platform (DATASUS), managed by the Brazilian Ministry of Health: https://opendatasus.saude.gov.br/dataset?groups=dados-sobre-srag
