# Supplementary material for: Time series analysis of comprehensive maternal deaths in Brazil during the COVID-19 pandemic
Source: Sci Rep. 2024 Oct 14;14:23960. doi: 10.1038/s41598-024-74704-x (PMC11471853; doi:10.1038/s41598-024-74704-x)
Supplement: Supplementary file 1 — Supplementary Material 1 [file 41598_2024_74704_MOESM1_ESM.docx]

**Time series analysis of comprehensive maternal deaths in Brazil during the COVID-19 pandemic; Supplemental Figures**

Mary Catherine Cambou^1*^, Hollie David^1^, Corrina Moucheraud^2^, Karin Nielsen-Saines^1^, Scott Comulada^1^, James Macinko^1^

1. University of California, Los Angeles, United States
2. New York University, United States

*Email addresses of authors:*

mcambou@mednet.ucla.edu*, hmdavid@mednet.ucla.edu, cm6512@nyu.edu, KNielsen@mednet.ucla.edu, WComulada@mednet.ucla.edu, jmacinko@g.ucla.edu

*Supplemental Figure 1. Holt-Winters forecast of predicted MMR compared to observed MMR in Brazil from 2008 to 2021.*

*
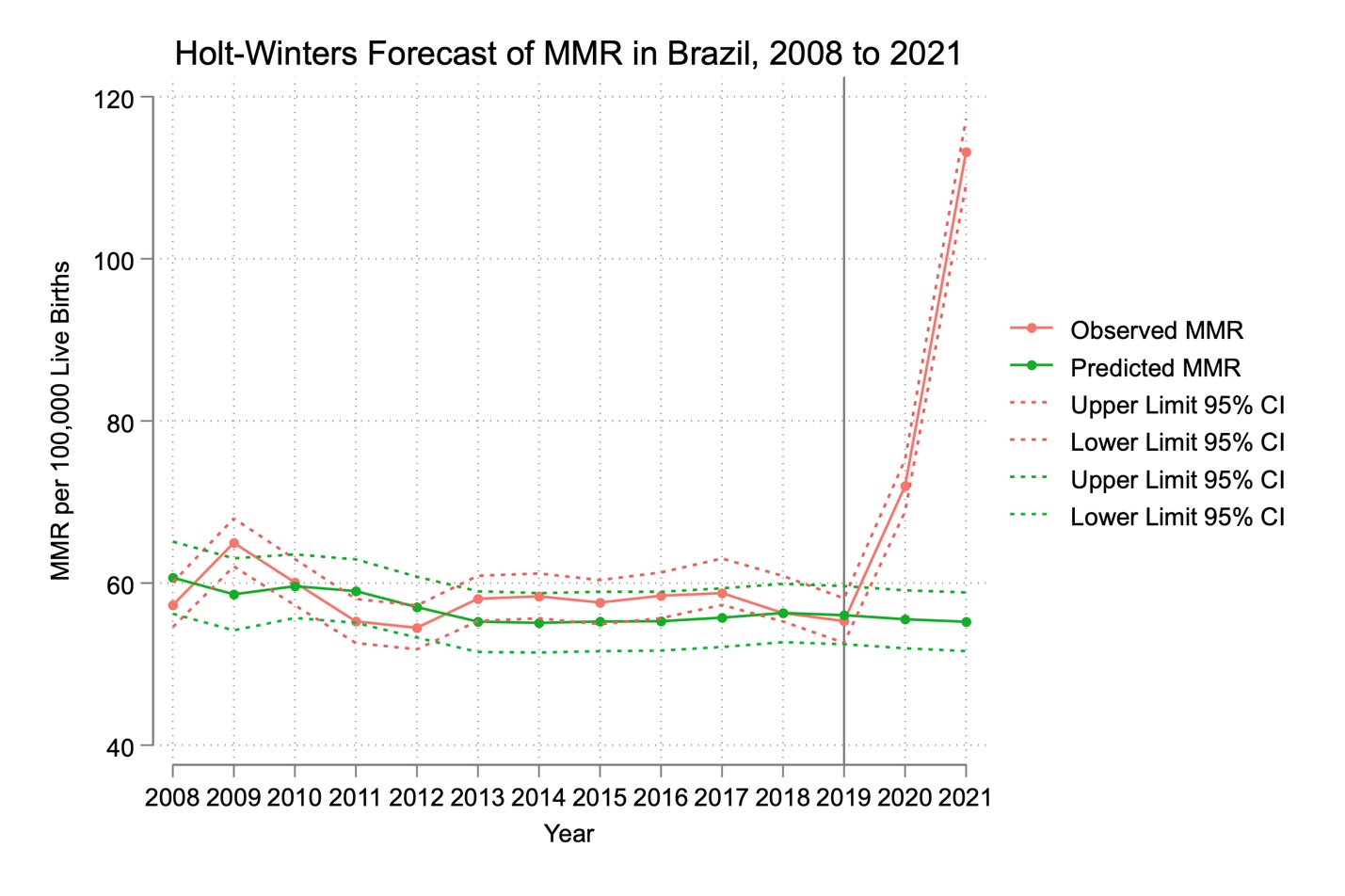
*

*Supplemental Figure 2. ARIMA forecast of predicted MMR compared to observed MMR in Brazil from 2008 to 2021.*

*
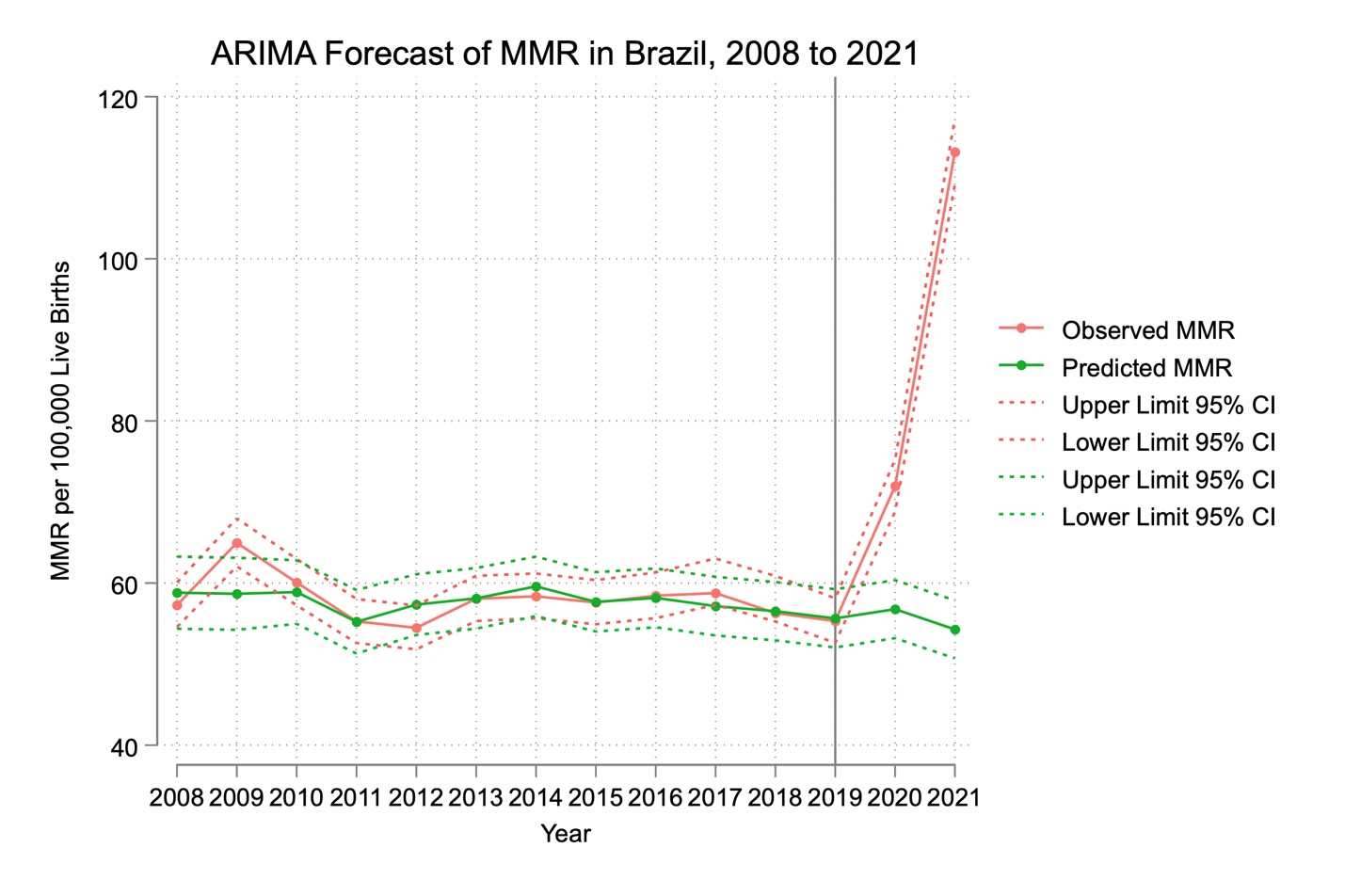
*
